# The reliability and validity of the SF-8 with a conflict-affected population in northern Uganda

**DOI:** 10.1186/1477-7525-6-108

**Published:** 2008-12-02

**Authors:** Bayard Roberts, John Browne, Kaducu Felix Ocaka, Thomas Oyok, Egbert Sondorp

**Affiliations:** 1 Conflict and Health Programme, Health Policy Unit, Department of Public Health and Policy, London School of Hygiene and Tropical Medicine, UK; 2 Health Services Research Unit, Department of Public Health and Policy, London School of Hygiene and Tropical Medicine, UK; 3 Faculty of Medicine, Gulu University, PO Box 166, Gulu, Uganda

## Abstract

**Background:**

The SF-8 is a health-related quality of life instrument that could provide a useful means of assessing general physical and mental health amongst populations affected by conflict. The purpose of this study was to test the validity and reliability of the SF-8 with a conflict-affected population in northern Uganda.

**Methods:**

A cross-sectional multi-staged, random cluster survey was conducted with 1206 adults in camps for internally displaced persons in Gulu and Amuru districts of northern Uganda. Data quality was assessed by analysing the number of incomplete responses to SF-8 items. Response distribution was analysed using aggregate endorsement frequency. Test-retest reliability was assessed in a separate smaller survey using the intraclass correlation test. Construct validity was measured using principal component analysis, and the Pearson Correlation test for item-summary score correlation and inter-instrument correlations. Known groups validity was assessed using a two sample t-test to evaluates the ability of the SF-8 to discriminate between groups known to have, and not have, physical and mental health problems.

**Results:**

The SF-8 showed excellent data quality. It showed acceptable item response distribution based upon analysis of aggregate endorsement frequencies. Test-retest showed a good intraclass correlation of 0.61 for PCS and 0.68 for MCS. The principal component analysis indicated strong construct validity and concurred with the results of the validity tests by the SF-8 developers. The SF-8 also showed strong construct validity between the 8 items and PCS and MCS summary score, moderate inter-instrument validity, and strong known groups validity.

**Conclusion:**

This study provides evidence on the reliability and validity of the SF-8 amongst IDPs in northern Uganda.

## Background

The 20 year war in northern Uganda between the government and a rebel group, the Lord's Resistance Army, has resulted in almost two million internally displaced persons (IDPs) being forcibly moved into government-established camps to reportedly protect the civilians and aid the government's counter-insurgency campaign against the rebels. These IDP camps are characterised by extreme over-crowding, high rates of mortality, morbidity, and insecurity [1-3].

International humanitarian standards note the need to provide a wide range of interventions to comprehensively address physical and mental health [4]. The ability to measure general physical and mental health amongst a conflict-affected population is important to help understand the overall health situation, detecting health variances between population sub-groups, determinants of health, and the impact of health-related interventions. Health-Related Quality of Life (HRQOL) instruments provide a useful means of measuring health outcomes at the population level and have been used with refugees repatriated to North America and Western Europe [5]. However, their use in conflict-affected environments has been restricted to assessing just one dimension of general health (social functioning) [6,7]. The HRQOL instruments used have also not been validated in conflict-affected environments. A brief, easily translatable, interviewer-administered HRQOL instrument could make an important contribution in measuring overall general physical and mental health in conflict-affected populations.

The SF-8 developed by QualityMetric is one potential instrument that meets criteria of brevity (it has a 1–2 minute administration time), ease of translation and use. The instrument provides a generic measure of physical and mental health status which is not specific to age, disease or treatment group. It can be interviewer-administered and so used with respondent groups with low literacy levels [8]. The instrument uses single-item scales addressing eight domains of general health, physical functioning, role limitations due to physical health problems, bodily pain, vitality (energy/fatigue), social functioning, mental health, and role limitations due to emotional problems. Physical and mental summary scores are produced and can be compared against well-developed norms in other populations [8].

The brevity of the SF-8 is achieved by losing precision compared to related longer instruments such as the SF-36 developed by the Medical Outcomes Study group which have multi-item scales [9]. However, the differences between the SF-8 and SF-36 are mitigated in population surveys where precision is achieved much more by drawing a larger representative sample than by increasing measurement reliability [8].

The SF-8 has been translated in over 30 different languages, and used in a number of countries [8,10-12]. Individual scales of related longer instruments such as the SF-36 have been successfully used with conflict-affected populations [6,7,13]. However, the reliability and validity of the SF-8 has not been demonstrated for use with populations affected by conflict. The purpose of this study was to test the validity and reliability of the SF-8 with a conflict-affected population in northern Uganda.

## Methods

This study formed part of a broader study investigating risk factors associated with general physical and mental health, and post-traumatic stress disorder (PTSD) and depression amongst IDPs in northern Uganda. Further details of the broader study can be found elsewhere [14,15].

### Survey questionnaire

The SF-8 was the selected HRQOL instrument. Criteria for selecting the health status instrument to be used in the questionnaire included the following: low burden to respondent and data collector; conceptual appropriateness; ease of translation and cultural adaptation; and established psychometric properties. Relevant published articles and internet sources were consulted to select the HRQOL instruments, [16-25] and other potential instruments were reviewed such as the SF-12; SF-36; EuroQol (EQ5D), Health Status Questionnaire (HSQ), and WHO Quality of Life Bref (WHOQOL Bref). It was decided that the SF-8 most closely met the selection criteria.

The questionnaire contained the 8 items of the SF-8, with a 4 week recall period. Each item has a 5 or 6 point response range. Physical (PCS) and mental (MCS) component summary measures were calculated by weighting each SF-8 item using a norm-based scoring method given in the instrument guidelines [8]. Higher summary PCS and MCS scores indicate better health. Scores above and below 50 are considered above and below the average in the general U.S. population [8].

The SF-8 was translated into Luo, the main language of Gulu and Amuru districts, using recommended guidelines [8,23,26,27]. This involved forward and back translation and a detailed review by the study team. Forward translation into Luo was conducted by a retired education lecturer at Gulu University. It was then back-translated into English by a staff member of Gulu University. Both translators were fluent in Luo and English and experienced in translation. A review of the back translation was conducted by the study team to ensure that the meanings and concepts of the questionnaire items remained. Two out of three members of the study team reviewing the translation were fluent in Luo and English. This was followed by pre-testing for accuracy of translation and also piloting the questions with a sample of IDPs. The pre-testing was conducted with 35 randomly selected respondents from an IDP camp not used in the main survey. The respondents were of a similar socio-economic status as all were displaced. A group review was held by the study team and data collectors used for the pre-testing to check for errors or problems. The data collectors were all fluent in Luo and English. A final forward and back-translation was then produced and a final review conducted by the study team. The piloting revealed that all the questions were answered, and there was a good distribution of answers from the questions, and the interviewers felt there was a clear understanding of the questions.

The survey questionnaire also included instruments to measure PTSD and depression. PTSD was measured using the original version of Harvard Trauma Questionnaire (HTQ), and depression was measured using the Hopkins Symptoms Checklist-25 (HSCL-25) [23,28]. The HTQ and HSCL-25 have been developed specifically for conflict-affected populations and have been widely used and tested for reliability and validity in a number of countries [6,7,13,23,28-34]. The HTQ and HSCL-25 are consistent with the *Diagnostic and Statistical Manual for Mental Disorders, Fourth Edition*[35] Both instruments use a recall period of 1 week. The HTQ and HSCL-25 produce mean scores for levels of PTSD and depression which can be dichotomised as meeting or not meeting symptom criteria of PTSD (scores ≥ 2.0) and depression (≥ 1.75) [27]. A multiple-response item was included on self-reported physical health conditions over the past 1 month (eg. fever/malaria, diarrhoea, respiratory infections, sexually transmitted infections). The survey questionnaire also had items on respondent demographic and socio-economic characteristics which were statistically tested for their association with PCS and MCS (the results are described elsewhere [15]). The questionnaire (including the HTQ and HSCL-25) was translated from English into Luo following the process described above for the SF-8 items.

### Study setting and participants

The study setting was Gulu and Amuru districts in northern Uganda. These districts contain an estimated 650,000 IDPs which is approximately 40% of all IDPs in Uganda. Up to 80% of the districts' population live in camps which range in size from 1,100 to almost 60,000 [36,37]. The study population was adult (≥ 18 years old) male and female IDPs. IDPs were defined as people living in the officially recognised IDP camps in Gulu and Amuru districts.

### Data collection

A cross-sectional survey design was followed using a multi-stage cluster sampling method [38]. The sample size calculation was determined based upon the requirements of the broader study noted above. The sampling frame was a list of the total population of IDPs living in all the 65 officially recognised IDP camps in Gulu and Amuru districts [37]. The first stage of the sampling was to randomly select the clusters from which the IDP camps would be selected. 32 clusters were chosen rather than the more common use of 30 clusters to reduce the design effect (a correction factor accounting for heterogeneity among clusters) which arises from cluster surveys. A higher number of clusters reduces the design effect. Therefore 32 clusters were selected rather than the more commonly used number of 30 clusters [39]. The clusters were selected and allocated to the IDP camps using the probability proportional to size technique [38]. The 32 clusters were allocated to 28 camps using this technique. The total population living in the 28 selected camps was 452,702. Due to the large population sizes of the selected camps, a second stage was used to randomly select administrative zones within the sampled IDP camps to act as individual clusters. The third stage consisted of randomly choosing individuals from the selected clusters. The Expanded Programme on Immunisation method was used to randomly select households for this stage and one individual was then randomly selected from the eligible individuals within the household [39-41]. A team of 15 data collectors was recruited for the survey (8 men and 7 women) who were all from the Acholi region of northern Uganda, spoke fluent Luo and English, and had experience of data collection in IDP camps in northern Uganda. Six days training was provided for the overall study. The data collection took place between 6 and 27 November 2006. The translated Luo questionnaire administered and each interview took between approximately 35 and 45 minutes. Two data entry clerks were used to enter the data into SPSS, version 14.0 (SPSS Inc, Chicago, USA).

In addition to the larger main survey, a separate smaller survey took place to measure test-retest reliability. The SF-8 questions (4 week recall period) along with the participant name, sex and age were collected. The sample size was determined with the aim of measuring the reliability coefficients for the PCS and MCS scores of the SF-8. This used the assumption that the reliability coefficients calculated in the smaller survey for PCS and MCS would be 0.8, and to be 95% certain that it was above 0.70 with a standard error of 0.05, a maximum sample size of 90 would be required [42]. The SF-8 test-retest survey was conducted in an IDP camp in Gulu district. Participants were randomly selected using the methods described above. The first round of data collection took place on 18 November 2006 and 91 questionnaires were completed. The second round took place on 25 November 2006 and the same questionnaire was administered to the same participant by the same data collector. Cross-checking of name, signature (where possible), age and attendance slip was conducted to try and ensure no replacements had entered the sample. 9 respondents from the first round were absent (5 men and 4 women) and so a total of 82 questionnaires were completed. Of the final 82 participants, 48 were women and 34 were men. The mean age of respondents in the smaller survey was 33 years with an age range from 18 to 68 years. All respondents were IDPs.

### Ethical approval and consent

Ethical approval for the whole study was provided by the Ugandan National Council for Science and Technology, Gulu University, and the London School of Hygiene and Tropical Medicine. A consent form was used to ensure informed consent and clarify that no direct benefit could be expected from participating in the study. All data collected was confidential, and anonymous (except for the smaller test re-test survey). As some of the questions were on mental distress, referral information for support on mental health was provided. One of the study team was a psychiatrist and one of the team leaders was a double trained Clinical Psychiatric Officer/Mental Health Nurse who could offer advice if required. Supervision and quality control were provided by the 3 members of the study team and 2 team leaders.

### Statistical analysis

Data quality was assessed by analysing the number of incomplete responses to SF-8 items. A large number of incomplete responses may suggest respondents found the question confusing, inappropriate or uncomfortable to answer. The number of missing individual SF-8 items was recorded, and also the number of respondents who did not complete at least half of the SF-8 items [43]. Questionnaires with 1 or more incomplete SF-8 items were excluded from further analysis on the validity and reliability of the SF-8.

The distribution of item responses of the SF-8 was evaluated by testing for aggregate endorsement frequencies. This requires that for instruments with around a 5 point response range such as the SF-8, any item with two or more adjacent response points showing less than 10% of the responses on aggregate are problematic [44].

Test-retest reliability in the smaller survey was measured to analyse the degree to which the questionnaire yields stable scores over a short period of time (assuming there is no underlying change). The intraclass correlation (ICC) test was used for test-retest reliability. An ICC below or equal to 0.40 was considered to show poor agreement, 0.41–0.60 a moderate agreement, 0.61–0.80 a good agreement, and 0.81–1.00 excellent agreement [45-47].

The construct validity of the main survey was explored to test whether the instrument measured the underlying attributes of physical and mental health [42,48,49]. This was firstly assessed by using principal component analysis to explore how responses on particular items cluster together to represent unique constructs. The methods for the principal component analysis followed those used by the SF-8 developers to allow comparison of the factor structure of the Luo and English versions [8]. The steps for the analysis were, firstly, to perform a principal component analysis without rotation. The correct number of components were then derived by using Cattell's scree test. The selected components were then rotated to orthogonal simple structure. These rotated components were then interpreted on the basis of their correlations with the SF-8 items. The results were analysed for strength of association between the items and the components. Thresholds for the strength of association between an item and the component were used to guide the analysis. These thresholds were based on those used for the hypothesised associations between an item and the component used by the SF-8 developers. These thresholds were for a weak association (r ≤ 0.30), a moderate to substantial association (r 0.30–0.70), and a strong association (r ≥ 0.70) [8]. The correlations between the items and PCS and MCS components were then compared with the hypothesised correlations. The variance explained (the percent of the total measured variance in the SF-8 items explained by the two principal components) was also analysed. The results of the principal component analysis were also compared with those from the general US population sample conducted by the SF-8 developers (4-week recall version) as the US sample is the validated norm for the SF-8 [8].

Construct validity was also assessed by examining convergent and discriminant validity using the Pearson Correlation Test [42,48,49]. Convergent validity seeks to show that the dimensions of an instrument correlate with other dimensions of that instrument or another instrument which theory suggests should be related to it. Discriminant validity seeks to show low correlations between those dimensions that are theoretically unrelated or weakly related constructs. Convergent and discriminant validity were tested by examining the correlations of items with the PCS and MCS summary scores, and then examining inter-instrument correlations between the SF-8 items and PCS and MCS summary scores with the HTQ and HSCL-25 which were used to measure PTSD and depression. *A priori *hypotheses about the directionality and magnitude of the correlations were made assuming that items more closely related to a common dimension would show a stronger correlation of ≥0.50 [50,51]. It was hypothesised that there would exist strong correlations between the PCS summary score and items 1–5 (general health, physical functioning, physical role limitation, bodily pain, vitality), and strong correlations between the MCS summary score and items 6–8 (social functioning, mental health, emotional role limitation). For the inter-instrument correlation, it was hypothesised that stronger correlations would exist between the MCS summary score and PTSD and depression scores than the PCS summary score. A low correlation was considered to be below 0.30, a moderate correlation between 0.30 and 0.60, and a strong correlation above 0.60 [51,52].

Known groups validity was also used to assess the ability of the SF-8 to discriminate between groups known to be clinically different [42,48,49]. A two sample t-test was used to measure known groups validity in the main survey to evaluate the ability of the instrument to discriminate between groups known to be different [42,48,49]. The difference in SF-8 summary scores was calculated between respondents who reported having had one or more of the most commonly reported physical health problems in the past 1 month (fever/malaria, respiratory infection, and diarrhoea) and respondents who did not report having any of these physical health problems in the past 1 month. It was hypothesised that the groups reporting physical health problems would record lower summary scores, particularly for PCS. Similarly, groups of respondents who met symptom criteria for PTSD (HTQ ≥ 2.00) and depression (HSCL-25 ≥1.75) were compared with those who did not. It was hypothesised that the groups with PTSD and depression would record lower summary scores, particularly for MCS.

Comparisons were also made with the results of general US population as these results are the validated norm for the SF-8 and so allows a meaningful comparison [8]. It was hypothesised that significant differences in the PCS and MCS scores should occur between the two population groups.

Statistical significance was assumed for *P *values < 0.05 for all tests. All statistical analysis was performed using STATA version 9.2 (Stata Corporation, College Park, Texas, USA) and adjusted for the clustered design.

## Results

The total number of completed individual interviews was 1206. The overall response rate was 94%. There were 44 absent individuals, and 22 non-consenting individuals, and 12 incomplete interviews. 60% of respondents were women. The mean age of respondents was 35 years, with an age range from 18 to 84 years. 91% of respondents were from the Acholi tribe. 77% were married or co-habiting, and 31% had never attended school.

The descriptive statistics from the main study for the PCS and MCS components and the individual items are presented in Table 1. The mean PCS score was 42.21 and mean MCS score was 39.27.

**Table 1:** SF-8 item and summary descriptive statistics (N = 1206)

**SF-8 item**		**Mean (SD)**	**Response option frequencies (%)**					
			**1**	**2**	**3**	**4**	**5**	**6**
1	General health	39.98 (7.44)	1.99	6.88	26.2	38.81	21.89	4.23
2	Physical functioning	44.11 (11.14)	35.42	29.68	10.28	13.76	10.86	-
3	Role – physical	41.44 (11.50)	30.18	24.96	14.10	16.91	13.85	-
4	Bodily pain	44.59 (11.33)	21.08	12.27	15.75	23.71	21.72	5.47
5	Vitality	42.57 (8.12)	2.32	14.76	39.39	37.64	5.89	-
6	Social functioning	44.46 (11.25)	38.64	18.24	15.84	21.14	6.14	-
7	Role – emotional	39.68 (10.92)	27.69	24.21	9.95	27.12	11.03	-
8	Mental health	40.50 (12.06)	21.64	19.24	12.44	34.41	12.27	-

	Overall PCS score	42.21 (11.93)						
	Overall MCS score	39.27 (12.83)						

Abbreviations: MCS, mental component summary; PCS, physical component summary; SD, Standard deviation

### Data quality

4 interviews (0.3%) had 1 missing SF-8 item, and 2 (0.2%) interviews contained incomplete responses to at least half of the SF-8 items. This suggests excellent data quality. The results of the sensitivity aggregate endorsement frequency to examine the response distributions for each item reveal acceptable sensitivity of the instrument with 7 out of the 8 items performing well (Table 1). The only exception was item one (general health) in which 9% of respondents were in response option 1 or 2.

### Reliability

The ICC test-retest reliability results from the smaller survey (N = 82) were 0.61 for PCS and 0.68 for MCS and so showed a good agreement between the two time periods.

### Validity

The principal component analysis found evidence for the existence of two constructs: physical and mental. The results of the correlations between the individual items and two components of PCS and MCS are presented in Table 2. The correlations generally confirm the hypothesised associations of the items with the PCS and MCS components. Items 1–4 were hypothesised to be more strongly associated with PCS and they all show strong associations (r ≥ 0.70) with PCS and generally weak correlation (r ≤ 0.30) with MCS. The items hypothesised to be more strongly associated with MCS (items 6–8) showed a strong correlation (r ≥ 0.70) with MCS and generally weak correlation (r ≤ 0.30) with PCS. As noted by the SF-8 developers, the item for vitality (item 5) has a stronger correlation with PCS and than MCS (unlike the longer SF-36 instrument). However, the correlation of the item on vitality (item 5) with MCS in this study was lower than hypothesised by the SF-8 developers.

**Table 2:** Principal component analysis of the SF-8 (N = 1206)

	**SF-8 Items**	Hypothesized association *		General US Population **		**Uganda IDP Population**	
		**Physical**	**Mental**	**Physical**	**Mental**	**Physical**	**Mental**
1	General health	+++	+	0.74	0.30	0.79	0.21
2	Physical functioning	+++	+	0.87	0.17	0.78	0.20
3	Role – physical	+++	+	0.85	0.28	0.79	0.28
4	Bodily pain	+++	++	0.75	0.21	0.78	0.32
5	Vitality	++	++	0.58	0.48	0.68	0.16
6	Social functioning	++	+++	0.53	0.70	0.34	0.70
7	Role – emotional	++	+++	0.42	0.77	0.22	0.85
8	Mental health	+	+++	0.07	0.91	0.16	0.88

**Variance explained †**				**72.3%**		**67.5%**	

Abbreviations: IDP; internally displaced person; MCS, mental component summary; PCS, physical component summary;

* Hypothesised association for general US population by SF-8 developers (Ware *et al*, 2001):

+++ Strong association (r ≥ 0.70)

++Moderate to substantial association (r 0.30 – 0.70)

+ Weak association (r ≤ 0.30)

** General US population data collected by SF-8 developers (Ware *et al*, 2001).

† Variance explained = percent of the total measured variance in the SF-8 items explained by the two principal components.

Table 2 also compares the study results with those of the general US population measured by the study developers. This comparison shows that the correlations of items 1–4 with the PCS and MCS components are generally quite similar between the two studies. The correlations of items 6–8 with the MCS component are also similar between the two studies, but less so for the PCS component. The results for the item on vitality (item 5) vary more substantially than the other items between the two studies, particularly for the MCS component. The results for variance explained are slightly lower for this study (67.5%) than the general US population study (72.3%).

Convergent validity results are presented in Table 3. These results show a generally strong convergent validity (≥0.50) of PCS-related items (items 1–5) with the PCS summary score, and MCS-related items (items 6–8) with the MCS summary score. Conversely, there are weaker correlations of PCS-related items (items 1–5) with the MCS summary score and MCS-related items (items 6–8) with PCS summary score, indicating discriminant validity.

**Table 3:** Item-summary score and inter-instrument correlations (N = 1206)

		**SF-8 item-summary score validity**		**Inter-instrument validity**	
**SF-8 Items**		**PCS**	**MCS**	**PTSD †**	**Depression ±**

1	General health	0.70	0.33	-0.24	-0.32
2	Physical functioning	0.82	0.19	-0.28	-0.32
3	Role – physical	0.89	0.26	-0.31	-0.35
4	Bodily pain	0.80	0.38	-0.34	-0.35
5	Vitality	0.55	0.37	-0.18	-0.24
6	Social functioning	0.41	0.63	-0.36	-0.38
7	Role – emotional	0.34	0.81	-0.41	-0.43
8	Mental health	0.19	0.94	-0.40	-0.43

	PCS summary score	-	-	-0.28	-0.32
	MCS summary score	-	-	-0.40	-0.43

Abbreviations: MCS, mental component summary; PCS, physical component summary; PTSD, post-traumatic stress disorder; SD, Standard deviation.

† PTSD=Harvard Trauma Questionnaire mean score ≥2.00.

± Depression = Hopkins Symptoms Check List-25 mean scores ≥1.75.

Table 3 also presents the results of the inter-instrument correlation for construct validity between the SF-8 items and PCS and MCS summary scores with PTSD (HTQ) and depression (HSCL-25). The results confirm the hypotheses, with individual MCS related items and the MCS summary score having moderate correlations with PTSD and depression (convergent validity), and the individual PCS related items and the PCS summary score having low/moderate correlations with PTSD and depression (discriminant validity).

Two sample t-test results of known-groups validity are presented in Table 4. These confirm the hypotheses that the groups reporting physical health problems (fever/malaria, respiratory infection, or diarrhoea), PTSD (HTQ =≥ 2.00), or depression (HSCL-25 =≥ 1.75) would record lower PCS and MCS scores (convergent validity) than those not reporting physical health problems, PTSD or depression (discriminant validity). The difference in the mean PCS scores between those with and without physical health problems, PTSD and depression was 10.79, 6.13 and 6.37 respectively. The difference in the mean MCS scores between those with and without physical health problems, PTSD and depression was 4.16, 8.49 and 9.60 respectively. As hypothesised, the difference in the means for PCS is larger than MCS for the physical health group comparison, while the difference in the means for MCS is larger than PCS for the PTSD and depression group comparisons.

**Table 4:** SF-8 Known Groups Validity Scores for SF-8 (N = 1206)

Variable/Group *	**N**	**SF-8 Means**	**[95% CI]**	**SD**	**t**
**Physical Component Summary (PCS)**					
Physical health in last month: §					
Physical health problem	828	38.83	[38.09–39.57]	10.84	16.03
Without physical health problem	378	49.62	[48.52–50.71]	10.83	
PTSD: †					
With PTSD	654	39.41	[38.49–40.32]	11.92	9.19
Without PTSD	552	45.53	[44.61–46.46]	11.08	
Depression: ±					
With depression	812	40.13	[39.31–40.95]	11.92	8.98
Without depression	394	46.50	[45.44–47.57]	10.77	

**Mental Component Summary (MCS)**					
Physical health in last month: §					
Physical health problem	828	37.97	[37.10–38.83]	12.68	5.71
Without physical health problem	378	42.13	[40.84–43.42]	12.71	
PTSD: †					
With PTSD	654	35.39	[34.42–36.35]	12.54	12.13
Without PTSD	552	43.88	[42.91–44.85]	11.60	
Depression: ±					
With depression	812	36.14	[35.29–36.98]	12.26	13.02
Without depression	394	45.74	[44.60–46.88]	11.51	

Abbreviations: CI, confidence interval; MCS, mental component summary; PCS, physical component summary; PTSD, post-traumatic stress disorder.

* P < 0.001(2-tailed) for all results between comparison groups.

§ Physical health problem in last month = respondents reporting the three main physical health conditions reported in the survey (fever/malaria; respiratory problems; diarrhoea).

† PTSD=Harvard Trauma Questionnaire mean score ≥2.00.

± Depression = Hopkins Symptoms Check List-25 mean scores ≥1.75.

Comparisons can also be made with known groups outside of the survey sample such as the general US population used to determine the norms for the SF-8[8] It was hypothesised that the SF-8 scores for the survey population would be lower than the general US population. The overall PCS and MCS score for IDP respondents was 42.21 (SD = 11.93) and 39.27 (SD = 12.83), compared to 49.20 (SD = 9.07) and 49.19 (SD = 9.46) for the general US population.

## Discussion

The study reports on the first ever investigation of the SF-8 with a conflict-affected population. The results suggest that the SF-8 could be used for population studies in conflict-affected areas.

### Data quality

The SF-8 showed excellent data quality with only 0.3% of respondents answering less than half of The SF-8 items, suggesting an extremely strong understanding of all of the translated SF-8 items. Acceptable item response distributions were observed with 7 out of the 8 items performing well. Item one (general health) had only 9% of respondents in response options 1 or 2. This shows that few respondents perceived their general health as excellent or very good which could be expected given the extreme conditions in which the study population were living. However, the distribution of responses was acceptable for other response point for item one and for the other items in the SF-8. This suggests that the SF-8 was able to capture the range of health responses with a conflict-affected population.

### Reliability

The test-retest ICC results of the smaller survey showed good reliability for PCS. However, the quite volatile situation of IDP camps meant health changes over time could have occurred over a 1 week period and so lowered the ICC results. A shorter retest period may therefore be preferable for measuring test-retest reliability among conflict-affected populations.

### Validity

The results for the principal component analysis provided strong evidence to indicate that items 1 to 4 principally measure PCS, and items 6–8 principally measure MCS, but that the item for vitality (item 5) correlates more strongly with PCS than MCS. This supports the findings of the developers of the SF-8 on the instrument's validity [8].

Item-summary score correlation coefficients revealed generally strong convergent and discriminant validity for the Luo version of the SF-8. The item for vitality (item five) showed a low correlation with MCS, and PTSD and depression. Vitality is a more general measure and evidence from studies on the SF-12 and SF-36 suggest it correlates with both PCS and MCS components, and the developers of the SF-8 note that the vitality item does tend to show a stronger association with PCS than MCS in the SF-8 [50,53]. However, the results in this study population suggest a very weak association of the vitality item with MCS. Further studies could investigate the validity of the vitality item.

The inter-instrument comparison between the SF-8 and HTQ and HSCL-25 also showed a correlation between the PCS and particularly MCS components with PTSD and depression (with the exception of the vitality item). Strong validity was particularly evident in the known groups validity test with reported physical and mental health conditions having a significant effect on PCS and MCS scores. This provides evidence on the ability of the SF-8 to correctly detect variances in health within conflict-affected populations.

### Limitations

The study had a number of limitations. The HTQ and HSCL-25 used for the inter-instrument construct validity tests have not been validated in northern Uganda. Evidence from the study published elsewhere suggests that the HTQ and HSCL-25 were able to detect significant differences between groups that evidence from other studies suggest would be different such as women compared to men, and persons that have experienced greater exposure to traumatic events [14]. The average response rates for the items in the HTQ and HSCL-25 in the study was 99.6% which suggests excellent data quality for the instruments in the study. The HTQ and HSCL-25 also showed strong levels of internal consistency reliability. The Cronback α was estimated at 0.86 for the HTQ and 0.83 for the HSCL-25, above the recommended minimum threshold level for internal reliability coefficient of ≥0.70 [14]. Another published study which used the HSCL-25 in the IDP camps of northern Uganda provides a Cronbach α score of 0.90 [33]. The HTQ and HSCL-25 have also been validated and used with conflict-affected populations in a range of cultural settings [23,28-31]. However, further validation work is required of the HTQ and HSCL-25 to evaluate the psychometric quality of the instruments for use with populations in northern Uganda. Another potential limitation is that the HTQ and HSCL-25 both use a one week recall period, whilst the 4 recall period of the SF-8 was used in the study. It is not known what influence the discrepancy in time frame may have had on the validity of the tests. However, respondent understanding of the different recall periods appeared clear. 30 other questions separated the SF-8 questions and the HTQ and HSCL-25 questions in the questionnaire so it was not expected that respondents were confused about the different recall period. The data collectors were also very clear about the recall period in their questioning and did not report any confusion on this recall period. Lastly, the study did not assess the responsiveness of the instrument to measure changes over time as this requires longitudinal data which was beyond the scope of this study.

## Conclusion

The SF-8's brevity and ease of use means it provides a feasible method of measuring general physical and mental health of conflict-affected populations. This study provides evidence on the reliability and validity of the SF-8 amongst IDPs in northern Uganda.

## Abbreviations

CI: Confidence Interval; HTQ: Harvard Trauma Questionnaire; HRQOL: Health-Related quality of Life; HSCL-25: Hopkins Symptoms Checklist-25; IDP: Internally Displaced Person; ICC: Intraclass Correlation; MCS: Mental Component Summary; PCS: Physical Component Summary; SD: Standard Deviation.

## Competing interests

The authors declare that they have no competing interests.

## Authors' contributions

BR, JB involved in the manuscript concept and design. BR, KFO, TO participated in the data collection. BR, JB conducted data analysis and review. BR, JB involved in drafting and reviewing the manuscript. KFO, TO, ES involved in reviewing the manuscript.
